# On Rheumatism, in Its Various Forms, and on the Affections of Internal Organs, More Especially the Heart and Brain, to Which It Gives Rise

**Published:** 1842-04

**Authors:** 


### Art. XII.

*On Rheumatism, in its various forms; and on the Affections of Internal Organs, more especially the Heart and Brain, to which it gives rise.* By Roderick Macleod, m.d., Physician to St. George's Hospital.—*London*, 1842. 8vo, pp. 164.

Dr. Macleod's extensive experience and long connexion with a large hospital have afforded him a wide field for the study of rheumatism, one of the most common and most distressing diseases among the lower clases in this country. The contents of the present work, he informs us, have been derived, in part, from nearly 400 cases of rheumatism treated by him in his public capacity. From this ample store of materials he has composed a treatise which will be found very useful by those who have not had much personal experience of this disease, and are but little acquainted with its literary history. We cannot help expressing our surprise, however, that with such materials to write on, the author has given us so very little that is original, and has supplied us with so few positive data for enabling us to controvert, corroborate, or verify the statements of his predecessors. Even if regarded as a compendium of the present state of our knowledge the work is very defective. Indeed, it can only be looked upon as a sketch of the history of rheumatism, a sketch too, of what is, in almost every particular, already familiar to all well-informed members of the profession.

It must not be understood from this, that we think lightly of Dr. Macleod's book. It certainly bears the stamp of a man of observation and experience, who is familiar with his subject; it contains many important observations and sound pathological views; and it inculcates, forcibly and clearly, methods of practice which are for the most part highly judicious. It is not, however, exactly the kind of performance we had expected from its author; and we think it is not nearly so good a work as he is capable of producing.

Such a work on rheumatism as would be a real boon to the profession, and a decided advance on the present state of our knowledge, will require, in spite of our English dislike of such a thing, to be of goodly dimensions and pregnant with details; and he who shall achieve the production of it must have a keen appetite for laborious observation, must be a wholesome and powerful digester of statistical facts, and have abundance of time withal to complete the elaboration of his materials. Without these appliances and means we expect from no man, however ample his opportunities or sound his judgment, a work of considerable importance on such a subject. The great range of the pharmaceutical expedients which have been, aud continue to be adopted for the cure of rheumatism, will prevent any reasonable man from anticipating, with much confidence, the discovery of a new remedial agent, whose potency and versatile virtues shall entitle it to supersede, to any great extent, the remedies with which we are already familiar; and as it was with no such anticipation that we opened the volume before us, we hold it to be no imputation against it, that it renounces all claim to novelty in this particular also. But, to say truth, we feel assured, and would be glad if the conviction were more prevalent than it is, that what is wanted in medicine is far less the discovery of untried methods of treating diseases than of discriminative canons for the proper use of those which we possess. In respect to rheumatism this appears to be peculiarly desirable, since no important disease has ever been treated so variously, and few have had so many remedies which have appeared in the experience of some practitioners to beeminently useful, and in that of others not less eminently inert or injurious.

In his first chapter, the author expresses his concurrence in the established opinion, that the exciting cause of rheumatism consists chiefly in atmospheric vicissitudes: he agrees with Chomel in acknowledging also the frequent influence of hereditary predisposition. He states what has been known since the time of Boerhaave at least, that males are more subject to it in the acute forms than females, and that early and middle life furnishes the greatest number of cases.

For the purposes of describing the phenomena conveniently, and of pointing out the appropriate treatment, Dr. Macleod conceives it necessary to divide rheumatic affections into five classes, without however, contending that the names are free from objection; viz., *rheumatic fever;* the *arthritic* or *capsular;* the *chronic* or *muscular;* the *neuralgic;* and the *periosteal* forms of rheumatism.

Rheumatic fever. The second chapter contains a description of the phenomena of rheumatic fever, or acute rheumatism. The usual account is given of the febrile state, and of the intensity, and fluctuations of the pain. The apparent limitation of the inflammation to the parts exterior to the synovial membrane, is specified as the circumstance which distinguishes the affection to which he confines the denomination *rheumatic fever*, from the *capsular* form of acute articular rheumatism. The aggravation of the pain during the night, which has been sometimes ascribed to increased warmth, is justly referred rather to "a nocturnal exacerbation which is experienced in this, in common with most other febrile diseases," and in support of this view the statement of Mr. Malcolmson is noticed concerning rheumatism in India, "that the natives suffer an aggravation of pain at night, although placed under circumstances which render any increase of temperature from without impossible, as their repose is taken in any corner where they can find space to lie down, and without going to bed at all." (p. 13.)

The joints which are the most frequently affected are, he believes, the following, in the order in which they stand; the foot and ankle, the knees, the hands, wrists, and shoulders; the elbow and hip joints more rarely. Dr. Macleod is also of opinion that rheumatism sometimes affects the *tunica albuginea testis*:

"Persons subject to rheumatism have complained of acute pain in one testicle, coming on suddenly, accompanied by increased heat, and by great tenderness to the touch, but without tumefaction; the symptoms shifting from one testis to the other, and at last disappearing as suddenly as they had come on, just in the manner we see rheumatism change from one joint to another." (p. 21.)

This is no new fact, a case of apparent rheumatism of the testes being noticed by Stoerck.

Of the anatomical results of the inflammation on the apparatus of the joints in this form of rheumatism, we have only the following meager statement:

"Opportunities very rarely present themselves of examining the joints during the actual existence of acute rheumatism, because patients very seldom die during such attacks; I have, however, in two instances, been able to examine the state of the parts primarily affected, but without finding any very striking results. The external redness had subsided in both cases, and the swelling had very much diminished, so that nothing appeared but a certain portion of serum, or serum and lymph, in the subcutaneous cellular membrane. The fibrous tissues were perhaps rather thicker than natural, but without redness; the synovial membranes were without any apparent participation in the disease." (p. 17.)

*The treatment* is detailed in the third chapter, and is introduced by the following observations:

"The more I see of rheumatism in its acute form, the more I am disposed to think that at its onset it is amenable to the same laws as other inflammations. When an attempt is made to enforce this argument, the most common answer is, that rheumatism is a 'specific disease,' and by this all its peculiarities as to pathology and treatment are supposed to be accounted for. But I suspect that this expression is often used without any very 'specific' meaning. If it be intended to intimate that rheumatism is not identical in its phenomena with various other inflammations, it is unquestionably very true; but the same observation holds good equally with regard to the inflammation of numerous other textures, as well as of those implicated in rheumatism. Pleuritis is manifested by different general symptoms, and different organic changes from bronchitis, and pneumonitis is in these respects different from both. Inflammation of the skin differs from that of cellular tissue, that of veins from both. . . . . I say that the difference between the inflammation of the fibrous textures in acute rheumatism, and that of any other texture which may be assumed as a standard of comparison, is not greater than between such standard and various other inflammations which might be named. Again, if by 'specific' be meant that there is anything in the exciting cause of the disease different from that of other inflammations, there is obviously no ground for the assumption." (p. 30.)

There is some truth in all this; yet, as regards the existence or non-existence of some peculiarity in rheumatic inflammation of fibrous textures greater than can be presumed to depend on peculiarity of tissue, the question is neither fairly stated, nor logically argued. The difference between the symptoms of the several other diseases which are contrasted, consists in the nature of the pain, the character of the products and of the fever, and the functional disorder of the organs which are immediately concerned; and in these particulars only can they, or any of them, if the proposition be confined to its proper terms, be justly compared to rheumatism. To maintain that any other phenomena that belong to the latter, do not entitle it to be considered as different from the others to a greater degree than might be anticipated from the kind of texture which it affects, is a mere petitio principii. Are we to make no account of hereditary predisposition, of the acid perspirations, of the urinary sediments, and of the shifting of the local symptoms from joint to joint? The same laxity of logic which would assign so great a scope to diversities in the symptoms of simple inflammation in different tissues, merely because different tissues, would suffice to prove the converse equally, viz., that a wide diversity in the symptoms is enough to establish a difference in the tissues which may be affected, and therefore that the peritoneum and the pleura are dissimilar textures. Bouillaud, who reasons on this point in much the same strain as Dr. Macleod, attempts to get rid of one embarrassing objection in the following way. Referring to the fluctuation of the symptoms of acute rheumatism from joint to joint, he explains it thus: "The influence of the exterior cause which has produced the rheumatism primitively, acts on a part hitherto respected, and in this case it is not, as is said, the rheumatism of one diseased joint which influences that which is healthy, but this latter coming to be strongly affected, the other is disengaged more or less, in conformity with the grand law of Hippocrates: duobus doloribus simul obortis non in eodem loco, vehementior obscurat alterum." If this grand law were in full operation in acute rheumatism, we apprehend that instead of the frequent and remarkable fluctuations which occur, the evil which had made an effectual lodgement first, would be more tenacious of its position, would keep in check the pretensions of another, and not yield so often at the first brush, like the warriors of the celestial empire. We may add, that we have seen reason to believe that the occurrence which Bouillaud denies does sometimes happen in rheumatism; that a diseased joint excites rheumatic symptoms in others. Thus we have known intense acute rheumatism of a knee-joint, attended by acute pains in the contiguous articulations of the ankle and hip, which ceased almost immediately on the more considerable affection of the knee being relieved by local remedies. If, according to Bouillaud and Macleod, acute rheumatism differs from other inflammatory diseases merely in being an affection of the fibrous tissues, we wonder whether we should not number among the causes of rheumatic fever the ancient operation of the rack. We offer this conjecture because Bouillaud objects to the argument against the doctrine in question which has been founded on the absence of the tendency to fluctuation of the symptoms when a single joint only has been injured by external violence. If mechanical injury of numerous joints be incapable of producing rheumatic fever, there must be two kinds of inflammation liable to affect those parts; and before this can be received as an unsound argument, on the ground that the causes are different, it must be shown that there is something nearly as different in the phenomena of pleurisy, or peritonitis, from wounds, and from cold, as there is in rheumatism from cold, and simple inflammation of the fibrous textures from mechanical injury.

Dr. Macleod's opinion of the nature of rheumatism, above quoted, is curiously limited by the following sentence: "But I would be understood as speaking at present of the early stage alone of acute rheumatism, because if this has been allowed to pass, various changes in the pathological condition supervene, requiring other and often dissimilar treatment." Dissimilar, we presume, from what is required in other inflammations. In what pathological condition do these various changes occur? In that of the system generally, or of the joints? It is manifestly not in any extraordinary product of the inflammation, for none such is known to be formed. It must be therefore a change in action or disposition of the part, or of this and the system generally. From this follows the strange doctrine, that the inflammation is in the early stage simple, and like other inflammations; a little later peculiar, and unlike them. These views appear to have been suggested by the results of the treatment at different periods of the disease; the author having found acute rheumatism amenable to bloodletting and the other ordinary means of subduing inflammation, when treated by these at the commencement, seems to have concluded that at this stage acute rheumatism differs not from other inflammatory diseases that yield to the same remedies. This is a mode of reasoning by no means uncommon; but, surely, very far from being correct. For if all inflammatory diseases that yield to the same remedies are to be deemed for *that* reason identical in their nature, for the same reason all other diseases that yield to the same remedies must be concluded to be similar—an absurdity which needs no illustration.

The best treatment for rheumatic fever, Dr. Macleod conceives to be the ancient practice of *bloodletting* and *purging*.

"In well-marked cases of rheumatic fever, within the first week of their onset, and in individuals of the average degree of robustness, from twelve to twenty ounces of blood may be abstracted with advantage, several successive times, in the course of five or six days. In judging of the propriety of repeating the venesection, the three points chiefly requiring to be attended to are, the state of the blood, the character of the pulse, and the effect produced upon the rheumatic pains." (p. 32.)

He does not think the existence of the buffy coat sufficient of itself to warrant a repetition of the evacuation; but a loose coagulum, without the buff, he esteems a sufficient reason for avoiding venesection, even though the attack be otherwise acute. When the pulse loses its bounding character after the first abstraction of blood, he has not found further depletion necessary. The cessation or very great diminution of the pains, he thinks, warrants the omission of further use of the lancet; and he would avoid pushing the depletion further if no relief whatever resulted from bleeding practised once, or at most twice.

The *purgatives* he has found to answer best are from three to five grains of calomel, administered at night, and senna and salts in the morning.

"This discipline ought generally to be repeated on several successive days; indeed, throughout the whole course of a case of acute rheumatism, the due evacuation of the bowels ought to be an indication never lost sight of; and in many cases where the attack is comparatively mild, this is the only form of depletion required." (p. 34 )

These recommendations are in the highest degree important for the speedy removal of the disease, whether mild or severe; and the custom of some practitioners, who avoid purging from fear of giving occasion to injurious exposure of the person, cannot be too strongly reprobated.

"By the joint use of the expedients above mentioned—namely, bleeding and purging—acute rheumatism in a certain number of cases may be cut short at once; that is, within two, three, or four days; and I have more than once seen it completely and permanently removed within twenty-four hours, even although ushered in with great severity, and attended by much swelling and redness of one or more joints. If it be objected that the proportion of such very rapid recoveries is small, I readily admit this; but I would ask in return, by what other means a like number of cures within a similar period can be effected." (p. 34.)

On turning to the tabular view of the results of this treatment, in a subsequent chapter, we find the following remarks on it.

"It thus appears, that of the 206 cases of rheumatic fever, 148 were discharged cured within a month; and when it is borne in mind that it was a rule rigidly enforced not to send out patients who have had acute rheumatism as soon as they cease to complain of pain, we may safely assert, that the number above specified was cured within three weeks; 110 within a fortnight, and 60 within eight days. In a small number, perhaps one-eighth of the whole, the disease was absolutely cut short at once within two, three, or four days, and in five or six cases it was (even though accompanied by much swellings and redness of one or more joints) completely arrested within 24 hours." (p. 153.)

These statements give us no exact idea of the duration of the disease, but only of the time the cases were under treatment; so that in estimating the duration of the cases we may dispense with the deduction made for their residence in hospital after the disease had ceased, which would probably not exceed the time that had elapsed between the date of the seizure and admission. Our estimate of the advantages of the treatment recommended need not, however, be influenced by these considerations, for others have doubtless founded their conclusions on grounds similar to those of Dr. Macleod. That the success of this treatment is unparalleled, by what has been experienced by others who have treated acute rheumatism, we must hesitate to admit. Dr. Hope, who confined his treatment to one bloodletting, and that only in some cases, followed by an evening dose of calomel and opium, and a morning purgative draught, found only exceptional cases to remain under treatment for more than a week; Dr. Corrigan mentions nine days as the average duration of the cases which he treated with large and repeated doses of opium; Bouillaud found from one to two weeks the usual duration of the disease, when treated by copious and repeated bloodletting alone; and Dr. Law, with bloodletting and colchicum, effected his cures mostly in a fortnight. Now the majority of Dr. Macleod's cases extended beyond the second week, even when the subtraction of a week is allowed for mere residence in hospitals after the cures were effected.

Of *opium* Dr. Macleod speaks in the following terms:

"The remedy, which in acute rheumatism stands next in importance to bleeding and purging, is opium. If by the means already mentioned the pain has not been very much mitigated within forty-eight hours, or I should rather say if any pain then remain, opium should be had recourse to in doses sufficient to give relief. . . . . On the average, two grains in the course of the twenty-four hours are sufficient; and I have been led to think that the solid answer better than the liquid preparations. How much opium may be taken with impunity, or even with advantage, so long as its usual effects are counteracted by the presence of pain, I am not prepared to say; but I have often given four, and sometimes six grains in twenty-four hours." (p. 35.)

In those limited quantities opium has long been given in this disease, but we believe without producing all the remarkable relief to suffering which a more liberal use of it is calculated to afford. We are indebted to Dr. Corrigan for reviving the practice of Pearson in respect to the administration of opium; and, howsoever the treatment may be varied in other respects, the physician who has had opportunities of witnessing the very striking decrease of suffering which follows almost immediately, the reception of a second or third considerable dose of opium in acute rheumatism, will rarely omit the administration of it in the way recommended by Dr. Corrigan. This physician states that he has known two hundred grains taken by one patient in fourteen days, and without narcotism. Ten, twelve, and even fifteen grains a day we have known to be taken without any injurious effect, and with much relief to pain. Notwithstanding what has been said to the contrary by the physician to whom we have just referred, the tendency to produce constipation is an objection in many instances to the practice in question. Constipation is not indeed an invariable consequence, but when it does occur it has appeared to us very materially to protract the disease when of a severe character originally. In order to derive the full benefit of opium in rheumatism, without the disadvantage alluded to, its use should be so far suspended from time to time as to allow of the bowels being freely evacuated every second day.

Of *calomel*, given so as to affect the mouth, Dr. Macleod has no good opinion. He has repeatedly seen the disease continue after the mouth has become affected; and we may add that the impregnation of the system with mercury has the disadvantage, besides, of bestowing a liability either to relapse of the acute disease, or to protracted pains.

Of *diaphoretics* and of *cinchona*, the author also expresses himself unfavorably. The former, however, have the testimony of general experience in their favour in recent and mild cases; and the latter has found more advocates than Haygarth. It is certainly one among the many anomalies of acute rheumatism that bark does sometimes, especially after a copious bloodletting, effect a speedy recovery.

Rheumatic affections of the heart. The fourth chapter, and the three which follow, are devoted to the affections of the heart which occur in acute rheumatism. Bouillaud receives his usual lecture on the folly of his claim to the merit of having first pointedly specified the connexion of acute diseases of the heart with rheumatism. The author next adverts to the error which has been often committed of ascribing the affections of the heart to metastasis, and asserts their not unfrequent occurrence even before the joints become affected, and their still more frequent coexistence with the disease in the joints, all of which points have been already fully insisted on by Hope, Bouillaud, and others. So little is Dr. Macleod inclined to ascribe these affections to metastasis, that he says,

"The result of my experience, indeed, would lead me to infer, that if an individual has any given set of joints affected with acute rhematism, such joints are more likely to be relieved if the disease attacks other external parts than if it fix upon the heart. . . . . I have no hesitation, however, in expressing my conviction that the pericarditis supervening during rheumatic fever may more correctly be regarded as an extension of the disease, than as a true metastasis." (p. 45.)

To the opinion contained in the first part of the quotation we must hesitate to subscribe in the absence of detailed proofs. The heart, it can scarcely be doubted, is obnoxious to acute rheumatism very much as a joint is, and if it be so esteemed, the various modes and circumstances in which it may become the seat of rheumatic inflammation may be easily and correctly understood. Sometimes in rheumatic fever a joint may become affected with mere pain, without any other evidence of the inflammation which is existing in others, and, as remarked by Andral and amply admitted by Bouillaud, a similar neuralgic or dynamic affection of the heart may occur, causing much temporary derangement of its action, but without ending in actual inflammation. Sometimes, too, apparently true rheumatism of the heart becomes translated to an external part, just as it may from one joint to another; one instance of which is recorded by Dr. Wells, and another in the Edinburgh Medical and Surgical Journal a few years back. And without doubt, sometimes, just as shifting rheumatism of the joints may become concentrated in one articulation, it may also in the fibrous, or fibro-serous textures of the heart. Metastasis, in the absolute and full sense formerly attached to the word, is not now considered by well-informed practitioners, as necessary to the establishment of rheumatism of the heart, though doubtless it may and does occasionally occur.

Dr. Macleod contests an opinion which has been sometimes maintained, that repeated venesection in acute rheumatism tends to increase the frequency of the affections of the heart. We confess that we have been inclined to concur with Dr. Alison in believing that it does. There will be no little difficulty in establishing either view of this subject, and such evidence as has yet been laid before the public is inadequate to form the ground of a decided opinion. The strongest which can at present be adduced is perhaps to be found in a comparison of the proportion of affections of the heart which occurs in the practice of Bouillaud, and of those, who like him, bleed largely, and of others who adopt a different course of treatment. Bouillaud expressly affirms that these affections occur in about one half of his cases of acute rheumatism, and he bleeds to a considerable amount daily during the first five days. Hope estimates the proportion in his practice as noticed above, at about one in twelve, and his observations were made on about 200 cases. Dr. Corrigan saw but one instance in numerous cases treated chiefly by opium. Dr. Macleod, whose treatment in respect to bloodletting more nearly resembles that of Bouillaud, has met with a large proportion of rheumatic inflammations of the heart. He tells us that in 226 cases of acute rheumatism (capsular rheumatism being excluded) he noticed pericarditis fifty-two times. In treating of acute capsular rheumatism he says "of a large number of cases of rheumatic pericarditis which have fallen under my observation, only two have occurred where the synovial membranes had been the seat of the disease," (p. 113,) and he gives in the table at p. 137, eighty-one cases of rheumatic synovitis. We have seen that bloodletting was liberally practised in the rheumatic fever, which yielded the large proportion of heart affections: while in the capsular form, he says of this remedy "general bloodletting, so useful in urgent cases of the former (rheumatic fever), is here rarely if ever required." (p. 125.) Now the 226 cases of rheumatic fever presented fifty-two instances of pericarditis, nearer one in four than one in five, as stated by the author; while the eighty-one cases of capsular rheumatism yielded only two instances of pericarditis, about one in twenty. He no doubt ascribes this circumstance of his cases of capsular rheumatism to a peculiarity in the disorder, owing to which the heart is less liable to be affected than in the other acute form; but is nothing to be ascribed to the difference of practice, seeing the conclusion to which the contrast of Bouillaud's practice with that of the other physicians points? It is at least a serious fact that of 226 cases treated by the depletory method nearly one in four should have had pericarditis. The proportion would still be considerable if the other eighty-one cases were added, namely, nearly one in five and two-thirds. It is worthy of notice, also, that pericarditis alone is the affection of the heart specified by Dr. Macleod; and no doubt if the number of cases of endocarditis, which occurs by no means rarely without pericarditis, had likewise been stated, the proportion of cases in which rheumatic inflammation of the heart occurred in his practice would have been considerably augmented. When we consider the great powers of observation possessed by Andral, Louis, and Chomel, their general aversion, at least formerly, to bloodletting, and their having so strangely overlooked the connexion of pericarditis with acute rheumatism, is there not some reason to suspect that Bouillaud has been indebted for his light on this connexion, in a very significant degree, to the great difference of his practice? We do not presume to give a decided opinion in the present state of our knowledge; but we cannot avoid the statement of our conviction, that the subject is in the highest degree worthy of being more thoroughly studied than it has yet been. To the reader who may be biassed by *à priori* considerations, and may view the connexion to which we advert as too unlikely to be worth the trouble of investigation, we would just suggest a consideration of the effect of large and repeated losses of blood on the irritability of the heart.

Of the symptoms of rheumatism of the heart, Dr. Macleod justly remarks that little reliance can be placed on any but those ascertained by the ear. He distinguishes the stethoscopic signs as usual into two kinds, those of attrition, occurring in the pericardial inflammation, and the blowing sounds or valvular murmurs. Of the former, he says that they have been attributed by some to dryness of the opposed surfaces of the pericardium, by others to the roughness caused by the concretion of effused lymph. He inclines to the latter opinion, and with justice, for we may notice that Dr. Stokes was able to limit in a fatal case, by the friction-sound, the spot at which the serous surfaces continued free from adhesion after other parts had become adherent; and in that spot the membranes were covered with lymph. Dr. Macleod's account of the valvular murmurs is as follows:

"In many cases it may be heard all over the region of the heart, but is usually most distinct at points corresponding to the aortic, and to the auriculo-ventricular valves of the left side; that is to say, that where the relative situation of the parts is not changed by previous disease, the blowing sound will generally be best heard by placing the ear, or the end of the stethoscope, at the lower part, and towards the left side of the third bone of the sternum, for the aortic, and a little further to the left for the mitral valve. When the blowing sound is heard with the first sound of the heart, and where it is perceived on placing the instrument over the carotid arteries, it is reasonable to conjecture that it depends upon disease having produced, in the aortic valves, certain changes which cause obstruction to the free exit of blood from the ventricle. If the blowing be contemporaneous with the second sound, and not heard over the carotids, it will probably be found to depend on disease at the orifice between the left auricle and ventricle." (p. 56.)

The errors contained in this passage cannot be passed over in silence. In respect to the rules for distinguishing valvular disease at the mouth of the aorta, there is inaccuracy in the statement which refers to the sound in the carotids. It is not at all necessary that a murmur should exist in these vessels in order to enable us to determine that disease exists at the aortic valves; nor is it by any means a matter of importance that the murmur should be best heard at the point which is specified. The occurrence of the new sound in its greatest degree of development in the course of the aorta is all that it is necessary to ascertain, in order not to render it "reasonable to conjecture," but to entitle us to conclude with certainty that the aortic valves are diseased. Then as to the means of distinguishing disease at the orifice between the left auricle and ventricle, we had imagined that it was a fact now well ascertained, and even familiar to physicians, that a murmur with the second sound is an extremely rare occurrence in consequence of such disease. Every one knows that a murmur with the second sound is not an uncommon circumstance in valvular disease, whether acute or chronic, but it occurs, as all recent writers on diseases of the heart agree in stating, (certain questionable or rare instances excepted) at the aortic orifice, owing to regurgitation. It is now a good many years since the late Dr. Hope, one of the best observers, pointed out the peculiarity which characterizes the regurgitant murmur, and it is questionable if any other ever exists, dependent on disease of the mitral valve. What he discovered and taught on the subject is well known to the profession at large as both easily recognized and singularly accurate, and constant as a ground of diagnosis; namely, that the murmur from this cause is heard the most distinctly over the body of the ventricle, and towards its apex. So universally are these truths known to all who practise auscultation, that we should have concluded Dr. Macleod to have fallen into the errors of which we have treated thus freely, in consequence of hurry or inadvertence, if similar inaccuracies did not occur in another part of his work. Some of Dr. Macleod's remarks on the character and varieties of the valvular murmur are not very intelligible, for instance, "its general character, where the valves have not been previously damaged so as to admit of regurgitation, is that of a simple bellows-sound, but sometimes it assumes a harsher character, and sometimes a distinctly musical note." (p. 57.) What are the changes which occur when the valves are so damaged as to admit of regurgitation? Do the harsher and musical tones occur when regurgitation takes place or not? And if the former, do they constitute more the general character of the sound than when no regurgitation takes place?

The observations which are made on the signs of effusion into the pericardium are far from being satisfactory. After stating that the cardiac region is liable to present an extended dulness on percussion in pericarditis with effusion, he observes,

> "It (the dulness) does not usually assist us in detecting the pericarditis, because, before the effusion can have proceeded so far as to produce an unequivocal augmentation in the surface over which the sound is dull, the disease in all probability will have sufficiently manifested itself by other signs. And again, in that form of pericarditis which attends acute rheumatism, the effusion is most frequently chiefly composed of lymph which, though it may be in quantity sufficient to produce embarrassment in the action of the heart, yet the mere increase of bulk thus produced is not usually to such an extent as to be satisfactorily judged of by percussion. A much more appreciable, and therefore more generally useful sign, in reference to sound afforded by the effusion which takes place into the pericardium in rheumatism, is the sound of the heart itself being observed to become progressively more feeble and more distant, while the general symptoms show that this is not dependent upon mere sinking of the vital powers." (pp. 58-9.)

We cannot go along with Dr. Macleod here: we have been accustomed to believe that a quantity of effusion, of whatever kind, too small to affect the sound on percussion morbidly, will not obscure the sounds of the heart, as Dr. Macleod supposes; nay, it constantly happens that the dulness on percussion is sensibly augmented before the fluid effusion is so abundant as to obscure the sounds or impulse of the heart; increased in intensity, as these commonly are, by the augmented energy of the heart's action. The difficulty experienced by physicians in perceiving, by means of percussion, the existence of a moderate effusion into the pericardium, often, we believe, depends on neglect of the best mode of practising the percussion: without the use of a pleximeter, it is very difficult to mark the limits of a natural or morbid dulness with accuracy; but when the assistance of that instrument is sought, a very moderate deviation from the average limits of a normal obscurity of sound is readily detected. The information which this instrument is the means of yielding is peculiarly valuable in enabling us to form, in many instances which might otherwise be perplexing, a tolerably accurate judgment as to whether the extended dulness results from effusion of fluid, or from increased volume of the heart itself of a chronic kind. Thus an extension of the dulness upwards is commonly more characteristic of fluid effusion than of enlargement of the heart. It is of great consequence, sometimes, that the physician should know these facts, because cases may come under treatment for the first time when the circumstances necessary for the production of the attrition sounds may have ceased, and others may occur in which they never have existed.

Dr. Macleod does not enter into any important details of the general symptoms which belong to pericarditis, with the exceptions of the peculiarity of expression often witnessed on the countenance, and the occasional occurrence of delirium. The latter is mentioned as having been noticed many years ago by Andral, and subsequently by Dr. Francis Hawkins, Dr. Latham, and Dr. Watson. The symptoms of cerebral disturbance may consist of anxious delirium or wild insanity.

"So far as I know, in all the cases hitherto recorded in which rheumatism of the heart has been attended by symptoms of inflammation of the membranes of the brain, the patients have died, and the encephalon has been found intact, or at least without any unequivocal evidence of inflammation. . . . I have reason to believe, however, that pericarditis may occur, accompanied by cerebral symptoms of the severest character, and still the patient recover under the persevering use of proper remedies." (p. 62.)

Two cases of recovery are detailed, the one having been bled once, blistered on the chest, and having taken calomel and opium with purgatives; the other is stated, more particularly, to have been ordered at first calomel and opium, and after the delirium occurred, three days subsequently, to have had a grain of opium every six hours, the calomel being omitted, because the gums had become sore. The narcotic was subsequently increased until half a grain of acetate of morphia was taken every four hours, until amendment took place, which it did on the sixth day from the commencement of the incoherence. The knowledge that delirium, convulsive twitchings, tetanic spasms, and coma, occurring in connexion with acute rheumatism, indicate pericarditis, is of much consequence in directing the attention of the physician to the true source of the cerebral disorder, the more common symptoms of pericarditis (the physical excepted) being usually obscured by the derangement of the nervous system. Symptoms resembling chorea have been noticed by Dr. Bright, as also occasionally occurring in connexion with pericarditis. Dr. Macleod concludes his notice of these symptoms of cerebral disease with the following remark :

"But there is another affection of the head, altogether different from the preceding, which, nevertheless, I am inclined to think has been confounded with it :—namely, a true inflammation of the brain or its membranes, connected with rheumatism. This, however, in all the cases of it I have ever seen, has arisen in connexion with rheumatism of the synovial membranes, and without any pericarditis. On the other hand, I have never known fibrous rheumatism affect the brain, except secondarily, (I believe functionally,) and after the heart had become implicated." (p. 67.)

The effects of rheumatism on the membranes and substance of the heart, both immediate and secondary, and the common signs of increased bulk of the ventricles, are discussed in too elementary and sketchy a style to demand particular notice. One statement alone appears to merit a passing observation.

"We may have the aortic valves perfectly healthy, and yet the left ventricle unusually muscular. I think it probable that in such cases the muscularity will be found to consist almost entirely of increased bulk of the columnæ carneæ, and that the mitral valve will be found thickened, or otherwise changed, in such manner as to have required more than usual effort to make it act. Such at least has been the case in several instances of this nature, which I have examined." (p. 81.)

This increased bulk of the columnæ carneæ he ascribes to their increased action, as they have in such cases "to act upon a stiff and unyielding valve." If the most probable opinion of the use of these columnæ be correct—namely, that they contract simultaneously with the rest of the ventricle, thereby to prevent the shortening of the contracting ventricle from giving scope for the valve being pushed by the pressure of the blood too much up into the auricle, we can scarcely understand how their action should be increased by any common rigidity of the valve. It appears more consistent with sound pathology to refer this hypertrophy of the columnæ to the operation of a general law, not of a mechanical but of a vital nature, which operates very extensively in producing hypertrophy of tissues. We have repeatedly noticed ex-eccentric hypertrophy of the ventricles succeed an attack of rheumatism, which had neither interfered with the action of the valves, nor interrupted the passage of blood through the orifices; and have been inclined to ascribe the occurrence to what has been termed the *nutritive irritation*, which is so apt to occur in tissues bordering on those in which chronic inflammation, or acute inflammation imperfectly subdued, had existed; and also in those tissues themselves which had been so inflamed. Examples of this are common in the stomach and intestines.

The treatment is briefly discussed, and after bloodletting, general and local, testimony is given to the efficacy of the common practice of administering mercurial preparations freely in the acute inflammations of the heart.

Capsular rheumatism. In the eighth, ninth, and tenth chapters, the peculiarities of capsular rheumatism are detailed.

"If being different in their seat, in their symptoms, in their terminations, in the affections with which they are complicated, and in their treatment, be sufficient to prove that a distinction ought to be made between two diseases, then is rheumatism affecting the synovial membranes essentially distinct from the form of rheumatism already considered. The seat of capsular rheumatism is in the lining membrane of the joints and bursæ of the tendons; being, in this respect, the same as gout, to which it is, in various respects, very closely allied. The parts most liable to its attacks are the feet and hands, where it is, for the most part, easily recognized by the enlargement of the joints; but the peculiar characters of the disease are, perhaps, most strikingly displayed when it attacks the knee." (p. 92.)

The local symptoms are next described, and the peculiarity of the shape of the affected joints, dependent on distension of the synovial capsule with fluid, pointed out. The circumscribed swelling thus produced affords a contrast to the smaller and more diffused tumefaction of the form of rheumatism already described.

"Nor are the histories of the two diseases less remarkable for their points of difference. The capsular rheumatism is infinitely more persistent—affecting, for the most part, several joints, but generally becoming more especially fixed in a limited number, to be ultimately completely localized, and in a certain proportion of them to produce permanent changes of structure, or even disorganization of the parts." (p. 94.)

After describing the common appearances of inflammation on the synovial membrane, and the increased quantity of synovia found in the joints when death occurs during the course of the acute disease—the distortions and nodosities, which result in cases which have been prolonged, or have frequently relapsed, the author proceeds:

"Another appearance which sometimes presents itself is peculiar: the surface of the cartilages of the joint seems as if sprinkled over, in spots of greater or less extent, with a white powder, or smoothly painted of the same colour. When we try to scrape this off, we perceive that it is not merely superficial, but pervades the substance of the cartilage. The form of this deposit is thus considerably different from that which takes place in acute and well-marked gout, wherein it usually exists in small, irregular, detached portions, or as a kind of exudation which may even escape externally. (pp. 93-6.)

These deposits in capsular rheumatism have been found, by Dr. Macleod, to consist, like those of gout, of lithate of soda, although Dr. Chambers found in several instances of old synovial rheumatism, that they consisted of carbonate of lime. Another change produced in this form of rheumatism is suppurative disorganization of the affected joint. Several cases are given in confirmation of the assertion, that such an effect may result from rheumatic inflammation. These, added to the cases published by Chomel, (who, however, does not recognize them as rheumatic,) Piorry, Bouillaud, Carswell, and Malle, fully justify the conclusion that suppuration does occasionally result from rheumatic inflammation of a joint.

The merit of having distinguished this from the other form of rheumatism is ascribed to Dr. Chambers; and though we find in the writings of Störck special reference to the rebellious nature of rheumatism with effusion into the joints, most of the other peculiarities of capsular rheumatism have been overlooked by more recent writers. That the distinction is both well founded and important in several particulars there can be no doubt. In regard to some of the characteristics specified in the quotations, every practical physician must have had occasion to remark with Bouillaud, that "in general, all other conditions being equal, the *fixity* and the obstinacy of acute articular rheumatism are in an inverse ratio to the number of the articulations affected." We may notice the statements of Chomel on some of these points also, as additional evidence of the propriety and importance of the distinction in question, so far as they go. "In certain individuals," he says, "there remain irradicable traces of the disease: tophaceous concretions for example; sometimes even a state of morbid and permanent flexion of the articulation, as if a sort of anchylosis. But this never, almost, happens after a first attack, nor especially in a case of general articular rheumatism. These alterations occur particularly after many relapses, after the passage of the disease into the chronic state, and above all in the case of partial articular rheumatism." (Leçons, t. ii. p. 252.) Partial articular rheumatism, as every physician knows, when of considerable intensity, is almost always of the capsular kind.

Dr. Macleod states that the capsular rheumatism "occurs in persons who are of a feeble or debilitated constitution; or if it appear in the more robust, it is generally after some considerable and continued source of mental or bodily exertion," (p. 108.) It is also found to supervene upon gonorrhœa, and other venereal affections, but with the latter almost exclusively, "where long-continued courses of mercury have been adopted." (p. 109.) While detailing the general symptoms he mentions that a case occurred to him in which the copious sweats deposited a pulverulent substance on the skin, which proved to be lithate of soda. Van Swieten also notices the occasional occurrence of pulverulent deposit on the skin in rheumatism.

When inflammation of internal parts occurs in connexion with capsular rheumatism, which is a somewhat rare occurrence, it is stated that such inflammation is different from that noticed in rheumatic fever, in being a true metastasis, and not to the heart, but to the pleura or membranes of the brain. "Nor are the immediate results of such internal inflammation less remarkable; for while very few, if properly treated, die during the first onset of rheumatism of the heart, the mortality holds a very high ratio where the synovial inflammation becomes transferred to the serous membranes either of the head or chest." (p. 114.) When the membranes of the brain become affected, Dr. Macleod believes that the occurrence took place much more frequently in the advanced than in the early stage; and in cases which present the disease in a fixed form in but one or two joints. The disease in the joints may continue stationary for weeks, or even months, and ultimately the swelling may diminish without any obvious cause; and "whenever, under such circumstances, any (even the slightest) head symptom presents itself, it must be viewed with the greatest suspicion." (p. 115.) It is unnecessary to enumerate the symptoms of cerebral disease which may occur in these circumstances, as they have nothing peculiar in them; the important point is to know that such symptoms occurring in such a connexion, are of the most serious import. Several illustrative cases are detailed, and in the examination of the parts within the head after death nothing was noticed of a kind different from what occurs after ordinary meningeal and cerebral inflammation.

The pleuritic complication which is apt to occur in capsular rheumatism happens in much the same circumstances as the other. The products in several instances of this kind of pleurisy which were witnessed by Dr. Macleod were serum and pus in much abundance. In one reflection connected with this subject Dr. Macleod is, we apprehend, not altogether correct. He speaks of the pleurisy which sometimes occurs in rheumatic fevers as a mere extension of inflammation from the pericardium, while that of the capsular rheumatism is stated to result from metastasis primarily to the pleura. Now it is quite certain that pleurisy sometimes occurs in rheumatic fever without any affection of the pericardium; and we do not know any reason to conclude that, when it coexists with pericarditis, it is dependent on extension of inflammation from the pericardium; on the contrary, the structure of the covering of the lung would induce us to presume that it would be liable to be attacked by rheumatic inflammation, independently of such extension, because it is truly a fibro-serous membrane, as originally maintained by Basin.

Dr. Macleod has not seen any other visceral diseases result from metastasis of rheumatism than those which he has mentioned, and they are doubtless rare. Andral has recorded a case of apparent metastasis to the peritoneum; and Recamier, we think, to the intestinal mucous membrane. Rheumatism of the uterus, of a very unequivocal kind, is also treated of by Dezeimeris.

Local bloodletting is in this form of rheumatism mentioned as more frequently required than general depletion. Colchicum is considered "the great remedy," internally. A drachm of the vinum colchici is recommended to be given in the course of twenty-four hours, the morning dose being combined with a saline purgative. The remedy is to be discontinued if there be, "1st, the cessation of the pain; 2d, a depressed state of the nervous system; 3d, the occurrence of purging; 4th, the disappearance of the lithates from the urine." Opium and calomel are not recommended. Iodide of potassium receives its due meed of praise, for its virtues in relieving pain, and otherwise greatly benefiting cases in which the acuteness of the attack has been subdued. He thinks doses of two grains three times a day secure, in the majority of cases, all the beneficial effects of the remedy. The affections of the brain, &c. are to be treated on general principles.

Of the capsular rheumatism it is said,

"According to the preceding table (of 81 cases,) capsular rheumatism would seem to be much more prevalent among men than women, (47 to 34;) much more equally diffused over the different periods of adult age than the form of the disease first described; and much more prone to affect persons under forty than genuine gout is. At the same time, it is more the disease of middle life than either rheumatic fever or muscular rheumatism; from forty to forty-five years of age, giving us twenty-two out of eighty-one cases or rather more than one fourth, which is a much larger proportion than holds good with respect to either of the others." (p. 158.)

Another difference between the capsular rheumatism and rheumatic fever is found in the duration under treatment.

"Of the cases of rheumatic fever, one half were discharged cured in three weeks; of the cases of capsular rheumatism, more than half remained in hospital at the end of six weeks." (p. 159.)

In the remainder of the work there is little that is particularly worthy of notice, and we conclude with the following account of the muscular variety of rheumatism.

"After the middle period of life, as we have seen above, the tendency to rheumatic fever progressively and rapidly diminishes. Not so with regard to the muscular form of the disease, which is spread more equally over the whole period of adult age: of the acute fibrous cases, only eight occurred between the ages of fifty and sixty, rather more than one twenty-eighth part of the whole number; whereas the same period gives nine cases of the more chronic muscular form, being very nearly one ninth of the whole." (p. 160.)

